# The role of mononuclear cell tissue factor and inflammatory cytokines in patients with chronic thromboembolic pulmonary hypertension

**DOI:** 10.1007/s11239-015-1323-2

**Published:** 2015-12-14

**Authors:** Minxia Yang, Chaosheng Deng, Dawen Wu, Zhanghua Zhong, Xiaoting Lv, Zhihua Huang, Ningfang Lian, Kaixiong Liu, Qiaoxian Zhang

**Affiliations:** Intensive Care Unit, First Affiliated Hospital of Fujian Medical University, Fuzhou, 350005 Fujian Province China; Division of Respiratory and Critical Care Medicine, First Affiliated Hospital of Fujian Medical University, Fuzhou, 350005 Fujian Province China

**Keywords:** Chronic thromboembolic pulmonary hypertension, Tissue factor, C-reactive protein, Tumor necrosis factor-α, Monocyte chemoattractant protein-1

## Abstract

Thrombosis and inflammation are two major factors underlying chronic thromboembolic pulmonary hypertension (CTEPH). Tissue factor (TF), C-reactive protein (CRP), tumor necrosis factor-α (TNF-α) and monocyte chemoattractant protein 1 (MCP-1) may play critical roles in the process of CTEPH thrombosis and pulmonary vascular remodeling. Ten patients with a confirmed diagnosis of CTEPH, 20 patients with acute pulmonary thromboembolism and 15 patients with other types of pulmonary hypertension were enrolled in this study, along with 20 healthy subjects as the control group. The immunoturbidimetric method was used to determine the plasma content of CRP. The plasma levels of TNF-α, MCP-1, and TF antigen were measured by an enzyme-linked immunosorbent assay, and TF activity was measured by the chromogenic substrate method. Percoll density gradient centrifugation was used to separate peripheral blood mononuclear cells from plasma. The level of monocyte TF mRNA was examined by reverse transcriptase-polymerase chain reaction. The correlations between all indices described above were analyzed. In CTEPH patients, the expression of CRP, TNF-α, and MCP-1 was significantly higher than that in controls (*P* < 0.05). The levels of TF activity, TF antigen, and TF mRNA in monocyte cells were increased in CTEPH patients when compared with control subjects, but only the TF antigen and TF mRNA levels were significantly different (*P* < 0.05). In CTEPH patients, levels of CRP, MCP-1, and TNF-α significantly correlated with the level of TF antigen in plasma. TF gene expression was increased in patients with CTEPH, suggesting that blood-borne TF mainly comes from mononuclear cells. TF expression significantly correlated with levels of CRP, TNF-α and MCP-1. These factors may play an important role in the development of CTEPH via the inflammation–coagulation–thrombosis cycle.

## Introduction

Chronic thromboembolic pulmonary hypertension (CTEPH) is the consequence of thrombus resolution failure after the establishment of thrombosis within the elastic pulmonary arteries [1]. CTEPH is a common variation of pulmonary hypertension (PH) and is associated with significant morbidity and mortality [2, 3]. Historical data indicate that if left untreated, CTEPH is associated with a poor 5-year survival rate ranging from 10 to 40 % depending on the patient’s pulmonary hemodynamics [3, 4]. The treatment of CTEPH includes pulmonary thrombus endarterectomy (PEA), balloon angioplasty, and medical therapy. The outcome of PEA surgery is favorable,and its mortality rates less than 3–5 % [5]. Patients who are not candidates for surgery may benefit from PH-specific medical therapy [6]. However, the effects of medical therapy are limited. Until recently, the pathophysiology of CTEPH has remained poorly understood [7].

The embolic hypothesis claims that CTEPH is the result of a single or recurrent pulmonary embolism originating from sites of venous thrombosis [8]. Tissue factor (TF) is the initiation factor of the extrinsic coagulation pathway and functions together with factor VII [9]. Studies have shown that increased TF expression plays a critical role in the process of thrombosis. There are some reports of a link between PH and TF expression [10, 11]; however, few of these reports discuss CTEPH and TF expression. In this study, our aim was to detect the mRNA levels of monocyte-derived TF and its role during CTEPH thrombosis and remodeling of the pulmonary vasculature.

The role of inflammation in CTEPH pathogenesis has been emphasized in recent years. Levels of inflammatory markers, such as C-reactive protein (CRP) [7, 12, 13], tumor necrosis factor-α (TNF-α) [14] and monocyte chemoattractant protein1 (MCP-1) [15, 16], may be elevated in the plasma and thrombus tissues of patients with CTEPH, which is in correlation with hemodynamic data. TF is often upregulated upon vascular injury, inflammation (e.g. lipopolysaccharides, interleukins, TNF-α, CRP, MCP-1), hypoxia, and other conditions [17]. However, the relationship between inflammatory markers (e.g. CRP, TNF-α, MCP-1) and TF in CTEPH remains unclear.

## Methods

### Study population

All procedures were approved by the Ethics Committee of the First Affiliated Hospital of Fujian Medical University and all patients provided written informed consent. In this study, 10 patients with CTEPH were recruited between June 2013 and October 2014. CTEPH is defined by the following observations after ≥3 months of effective anticoagulation therapy [18]: (1) pulmonary arterial pressure (mPAP) >25 mmHg with a pulmonary capillary wedge pressure ≤15 mmHg; and (2) at least one (segmental) perfusion defect detected by ventilation and perfusion (V/Q) lung scan [19], multidetector computed tomographic (CT) angiography, or pulmonary angiography. If the V/Q scan were the primary mode of diagnosis, the results would need to be interpreted as a high probability of thromboembolic disease in order to be considered a positive test for CTEPH. A negative CT angiogram was not considered sufficient to exclude CTEPH. At the same time, 20 patients with pulmonary thromboembolism (PTE) and 15 patients with non-thromboembolic PH were enrolled in this study. We also enrolled a control group (n = 20) that consisted of healthy blood donors and was defined by the following observations: (1) healthy subjects who had a physical examination in the medical center of the First Affiliated Hospital of Fujian Medical University in the same time period, and (2) the physical examination was normal. The mPAP of CTEPH and non-thromboembolic PH patients was determined by right heart catheterization based on the recent consensus paper [20].

### Sample collection

Eight milliliters of blood were collected from each study subject (of note, no drugs that affect coagulation status were taken by subjects before sample collection) in two anticoagulant heparin and sodium citrate (1:9) vacutainers. One vacutainer was centrifuged at 3000 rpm for 15 min within 30 min of collection. The blood plasma was frozen at −80 °C for analysis of levels of CRP, TNF-α, MCP-1, and TF antigen and TF activity within 2 months. Blood monocytes were isolated by the percoll density gradient centrifugation method for measuring TF mRNA levels in monocytes.

### Miscellaneous

Plasma CRP levels were measured by the immune turbidimetric assay using the automatic dry biochemical analyzer (Johnson & Johnson, China) according to the manufacturer’s protocol. Levels of TNF-α, MCP-1, and TF antigen were determined by enzyme-linked immunosorbent assay (ELISA) (CUSABIO, China). The activity of TF was measured by the chromogenic substrate method (ab108906, Abcam, England).

### Primer design and RT-PCR

Blood monocyte samples were treated with Trizol reagent (Invitrogen, USA) and RNA was extracted in accordance with the manufacturer’s instructions. M-MLV reverse transcriptase was purchased from Thermo Scientific. The purity of the RNA was analyzed by OD260/OD280 ratio measured by ultraviolet spectrophotometry. RNA samples with an OD260/OD280>1.8, indicating high RNA purity, were used for the following experiments. An aliquot of RNA underwent gel electrophoresis at 80–100 mV.

Primer pairs were designed for TF and β-actin (internal control) using their DNA sequences. Primers were synthesized by Shanghai Biotechnology (China). The primers for TF were: forward 5′-TAC TTG GCA CGG GTC TTC TC-3′ and reverse 5′-TGT CCG AGG TTT GTC TCC AG-3′, resulting in a 119 bp product. The primers for β-actin were: forward 5′-CGG GAA ATC GTG CGT GAC-3′ and reverse: 5′-TGG AAG GTG GAC AGC GAG G-3′, resulting in a 434 bp product. Cycling conditions were as follows: 95 °C denaturation for 5 min; 40 cycles of 95 °C for 45 s, 58 °C for 30 s, and 72 °C for 30 s; and a final 10 min 72 °C extension. Products underwent 2 % agarose gel electrophoresis containing 0.5 μg/mL ethidium bromide and a gel imaging system (HBMA-9600, China) was used to visualize and photograph the results with β-actin as the internal reference. TF expression was semi-quantitatively determined by the imaging software.

### Statistical analysis

Statistical software SPSS17.0 was used for statistical analysis. Quantitative data were expressed as mean ± standard deviation (SD). Multiple groups were compared by analysis of one-way ANOVA. Coefficients of correlation were calculated by the Spearman rank test. Probability values (two-sided) were considered significant at a value of *P* < 0.05.

## Results

### Characteristics of the study population

A total of 65 subjects were included in this study: 10 patients with CTEPH, 20 patients with PTE, 15 patients with non-thromboembolic PH, and a control group of 20 healthy subjects. Non-thromboembolic PH cases included idiopathic PH (n = 5), familial PH (n = 2), and PH associated with respiratory disease (n = 3), connective tissue disease (n = 2) or congenital heart disease (n = 3). Patient demographics and clinical characteristics are reported in Table 1. D-dimmer plasma concentrations were elevated in PTE, non-thromboembolic PH and CTEPH when compared with the control group, but only the difference between the PTE and control groups was statistically significant (*P* < 0.01). Compared with the control group, fibrinogen plasma concentrations were significantly elevated in the PTE and CTEPH groups at the time of diagnosis (*P* < 0.01). No significant differences in fibrinogen concentrations were detected between non-thromboembolic PH and control subjects. The above data suggest that the PTE and CTEPH patients are in a hypercoagulable state.

**Table 1:** Characteristics of the study population

	CTEPH (n = 10)	PTE (n = 20)	PH (n = 15)	Control (n = 20)
Age (years)	58 ± 9	52 ± 16	52 ± 21	51 ± 10
Sex (male/female)	3/7	9/11	6/9	12/8
Smoking history				
(Yes/no)	2/8	3/17	3/12	5/15
(Current/past)	1/1	1/2	1/2	3/2
mPAP (mmHg)	79.7 ± 21.19	ND	89.1 ± 11.2	ND
D-dimmer (mg/L)	1.08 ± 1.51^△^	5.04 ± 2.87^**^	1.12 ± 1.61^n.s.^	0.18 ± 0.08
Fibrinogen (g/L)	4.40 ± 0.56^**▲^	5.18 ± 1.19^**▲^	2.82 ± 0.88^n.s.^	2.67 ± 0.43

*CTEPH* chronic thromboembolic pulmonary hypertension, *ND* not done, *PH* pulmonary hypertension, *PTE* pulmonary thromboembolism, *mPAP* mean pulmonary arterial pressure

**P < 0.01 and n.s indicates not significant (*P* > 0.05) versus control group. ^△^ *P* < 0.01 versus PTE group and ^▲^ *P* < 0.01 versus non-thromboembolic PH group

### CRP, MCP-1, and TNF-α levels in CTEPH

**Fig. 1:**
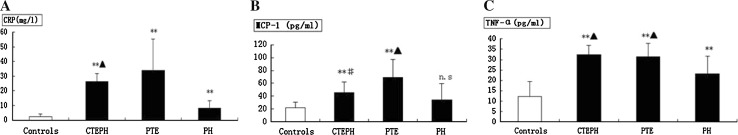
CRP, MCP-1 and TNF-α levels in CTEPH. **a** Plasma level of CRP in CTEPH (26.44 ± 5.18 mg/L, n = 10), PTE (33.83 ± 21.47 mg/L, n = 20), and non-thromboembolic PH (8.35 ± 5.03 mg/L, n = 15) patients and in control subjects (2.42 ± 1.71 mg/L, n = 20). **b** Plasma level of MCP-1 in CTEPH (45.49 ± 16.52 pg/mL, n = 10), PTE (69.37 ± 27.58 pg/mL, n = 20), and non-thromboembolic PH (34.20 ± 25.55 pg/mL, n = 15) patients and in control subjects (22.27 ± 8.59 pg/mL, n = 20). **c** Plasma level of TNF-α in CTEPH (32.34 ± 4.53 pg/mL, n = 10), PTE (31.26 ± 6.62 pg/mL, n = 20), non-thromboembolic PH (23.23 ± 8.44 pg/mL, n = 15) patients and in control subjects (12.12 ± 7.40 pg/mL, n = 20). Plasma concentrations are expressed as mean ± SD. ^**^ *P* value <0.01 and n.s indicates not significant (*P* > 0.05) versus control group. ^#^ *P* < 0.05 versus PTE group and ^▲^ *P* < 0.01 versus non-thromboembolic PH group

**Fig. 2:**
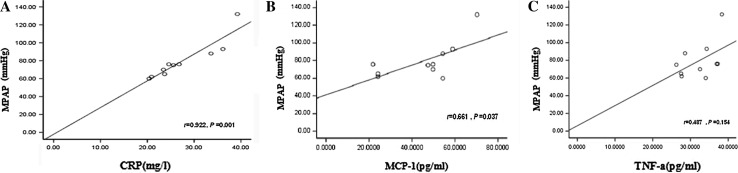
Correlation between mPAP and CRP, MCP-1, and TNF-α in CTEPH patients. **a** Correlation between mPAP and plasma level of CRP. **b** Correlation between mPAP and plasma level of MCP-1. **c** Correlation between mPAP and plasma level of TNF-α

As shown in Fig. 1, CRP and TNF-α levels increased significantly in all groups (CTEPH, PTE, and non-thromboembolic PH) compared with the control group (*P* < 0.05). In the CTEPH and PTE groups, the increase in the level of MCP-1 was significant (*P* < 0.01). In the CTEPH group, the mPAP ranged from 62 to 132 mmHg, with a mean ± SD of 79.7 ± 21.19 mmHg. Moreover, in CTEPH patients, both CRP (r = 0.92, *P* < 0.01) and MCP-1 (r = 0.66, *P* < 0.05) levels significantly correlated with pulmonary artery systolic pressure, while TNF-α levels did not (r = 0.49, *P* > 0.05) (Fig. 2), suggesting that there is a link between CRP and MCP-1 and severity of disease in CTEPH.

### TF levels in CTEPH patients

**Fig. 3:**
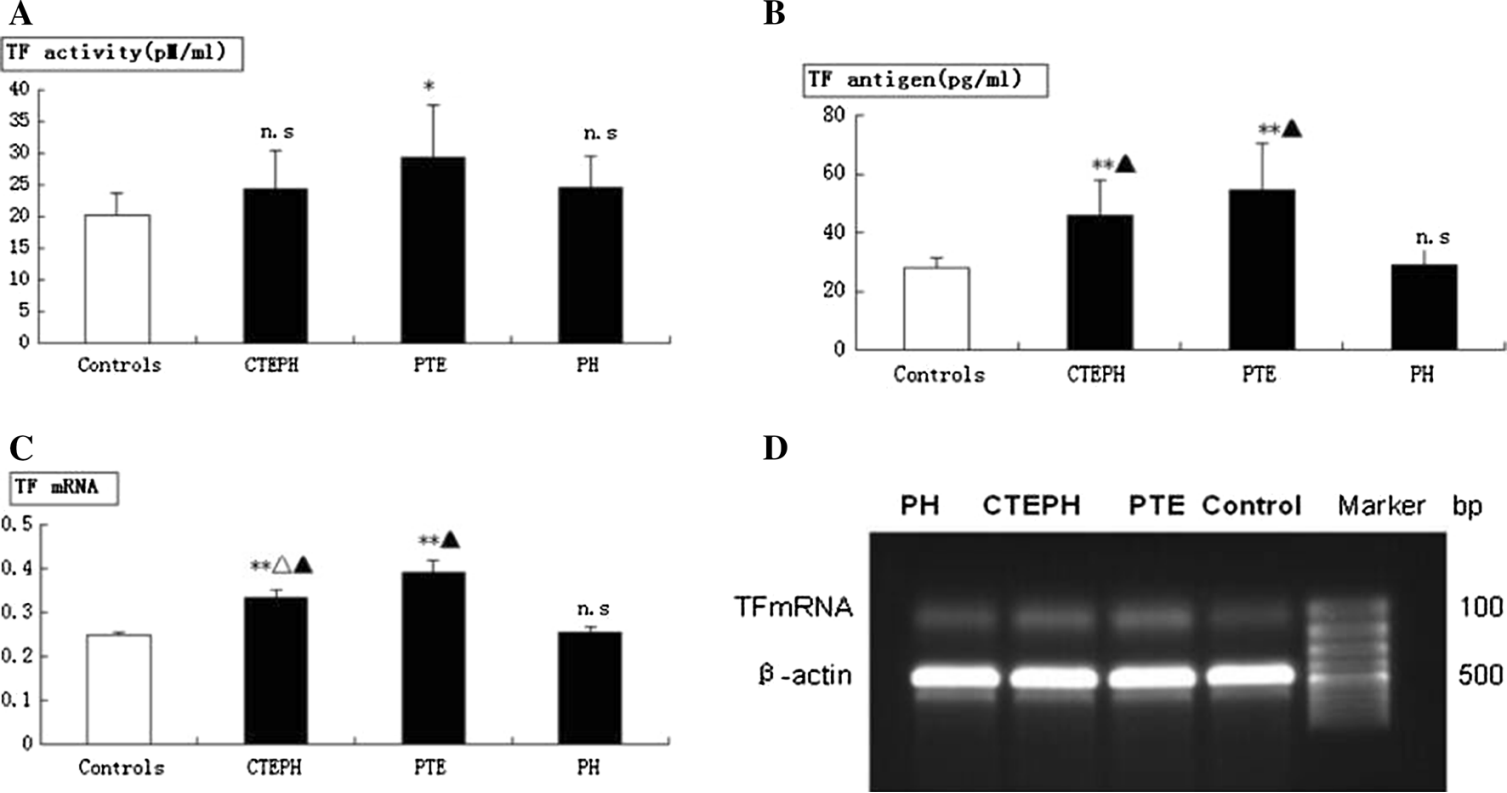
TF level in CTEPH patients. **a** Plasma level of TF activity in CTEPH (24.35 ± 6.10 pM/mL, n = 10), PTE (29.35 ± 8.26 pM/mL, n = 20), and non-thromboembolic PH (24.41 ± 5.20 pM/mL, n = 15) patients and in control subjects (20.24 ± 3.29 pM/mL, n = 20)**. b** Plasma level of TF antigen in CTEPH (45.68 ± 12.06 pg/mL, n = 10), PTE (54.68 ± 15.98 pg/mL, n = 20), and non-thromboembolic PH (29.11 ± 5.55 pg/mL, n = 15) patients and in control subjects (27.92 ± 3.33 pg/mL, n = 20). **c** TF mRNA expression in monocytes in CTEPH (0.33 ± 0.019, n = 10), PTE (0.39 ± 0.027, n = 20), and non-thromboembolic PH (0.26 ± 0.01, n = 15) patients and in control subjects (0.25 ± 0.01, n = 20). **d** RT-PCR products of TF from monocytes by gel electrophoresis. Plasma concentrations are expressed as mean ± SD. **P value <0.01, *P < 0.05 and n.s indicates not significant (*P* > 0.05) versus control group. ^△^ *P* < 0.01 versus PE group and ^▲^ *P* < 0.01 versus non-thromboembolic PH group

**Fig. 4:**
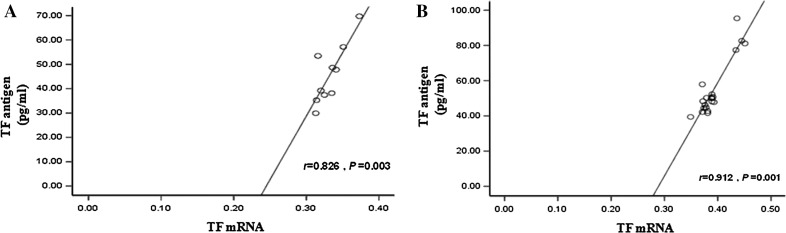
Correlation between TF antigen and monocyte TF mRNA levels. **a** Correlation between TF antigen and monocyte TF mRNA in patients with CTEPH. **b** Correlation between TF antigen and monocyte TF mRNA in patients with PTE

**Fig. 5:**
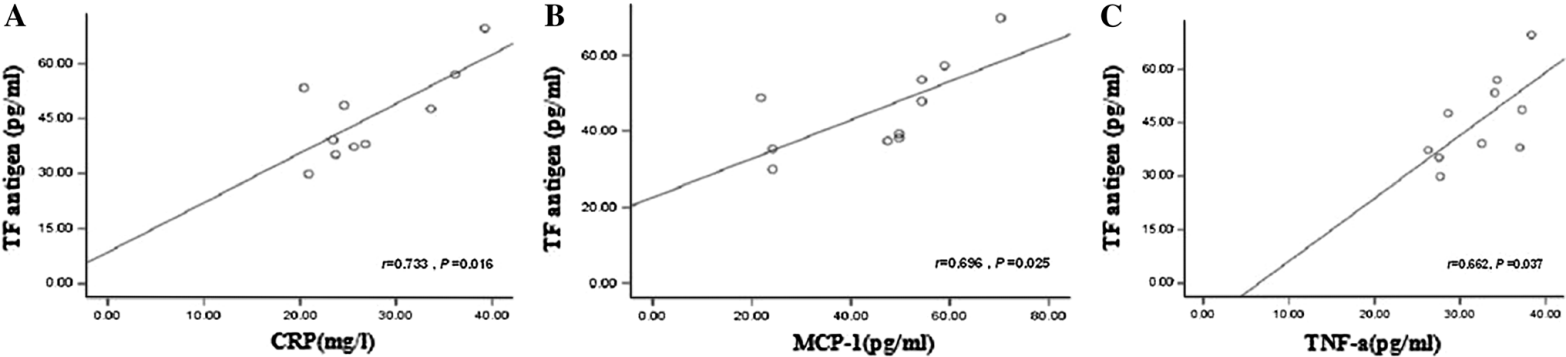
Correlation between TF antigen and CRP, MCP-1, and TNF-α levels in patients with CTEPH. **a** Correlation between TF antigen and plasma level of CRP. **b** Correlation between TF antigen and plasma level of MCP-1. **c** Correlation between TF antigen and plasma level of TNF-α

In this study, the activity of plasma TF increased in all groups (CTEPH, PTE, and non-thromboembolic PH) when compared with the control group, but only the PTE group resulted in a significant difference (*P* < 0.05). In CTEPH and PTE groups, the level of the TF antigen increased significantly (*P* < 0.01). TF mRNA levels in monocyte cells were significantly different in both CTEPH and PTE groups compared with the control group (*P* < 0.01). There also was a significant difference in TF mRNA levels between CTEPH and PTE groups compared to non-thromboembolic PH patients (*P* < 0.01; Fig. 3). Moreover, in the CTEPH and PTE groups, TF antigen levels significantly correlated with monocyte TF mRNA levels (r = 0.83, *P* < 0.01; r = 0.91, *P* < 0.01), suggesting monocyte TF may play a key role in CTEPH thrombosis and remodeling of the pulmonary vasculature (Fig. 4). Finally, in CTEPH patients, CRP (r = 0.73, *P* < 0.05), MCP-1 (r = 0.70, *P* < 0.05) and TNF-α (r = 0.66, *P* < 0.05) levels significantly correlated with plasma levels of TF antigen (Fig. 5), suggesting that these factors may promote the expression of TF antigen in monocytes and exacerbate thrombosis, thereby affecting the pathological process of CTEPH.

## Discussion

### TF hypercoagulability leading to thrombosis

TF is also known as factor III and it can initiate extrinsic coagulation by binding to and activating coagulation factor VIIa. The resultant TF-VIIa complex then activates factor X (factor Xa), triggering the generation of thrombin, which converts soluble fibrinogen into insoluble fibrin [21, 22]. Under normal circumstances, blood mononuclear cells and endothelial cells do not express TF [23]. It has been shown that the level of circulating TF in the blood of healthy donors is extremely low and does not appear to contribute to hemostasis [24, 25]. This suggests that increased TF levels and activity in the blood may trigger thrombosis [26].

Some reports have suggested that an underlying hypercoagulable state may be responsible for the development of CTEPH. We found increased TF activity in PTE patients, but not in CTEPH or PH patients, manifesting the hypothesis that thrombosis is associated with TF activity. However, TF activity does not always correlate with TF gene expression because TF activity may be affected by the change in expression of tissue factor pathway inhibitor (TFPI) or other anticoagulants or by anticoagulant therapy. In the present study, we have demonstrated the higher plasma level of TF antigen and activity in CTEPH patients compared with healthy subjects. It is also interesting that only the level of TF antigen in plasma is significantly increased while the level of TF activity is not. Burley argued that anticoagulants (e.g. low molecular weight heparin, heparin, TFPI, antithrombin III) can reduce TF expression and activity [17]. As we know, many patients with CTEPH often undergo anticoagulation therapy before diagnosis. Our results also illustrate this point as the expression of the TF antigen and TF mRNA levels increased in CTEPH patients but with no corresponding increase in TF activity, likely due to the impact of anticoagulant therapy.

### Tissue factor closely related with monocytes

Monocytes were the first cell type identified that can synthesize and express TF. TF is also present in other cell types that associate with monocytes. Our study showed that the levels of monocyte TF mRNA in circulating blood increased in all groups (only CTEPH and PTE groups showed a significant difference) when compared to the control group, with a concurrent increase in the levels of TF antigen and activity. This suggests that TF expression in monocytes can play an important role in the pathogenesis of PTE and CTEPH. Furthermore, we have found that the expression of the TF antigen significantly correlates with monocyte TF mRNA levels in both CTEPH and PTE patients, suggesting that levels of TF antigen are closely related to TF mRNA levels in monocytes under pathological conditions. Therefore, the results indicate that mononuclear cells are the source of blood-borne TF, which plays a key role in the process of physiological hemostasis and thrombosis [27].

In this study, we observed that the increase in expression of TF in monocytes can result in the formation and maintenance of thrombus and may play an important role in the development of PTE and CTEPH. Zhang et al. have shown TF involvement in the pathophysiological process of PTE, and that high levels of TF expression in pulmonary arterial tissue adjacent to emboli could lead to an increase in local coagulation activity [9]. Kooiman et al. have also suggested that it is indispensable to assess the relationship between TF and acute/chronic thromboembolic disease [28].

### Coagulation–inflammation–thrombosis circuit

Our observations raise the following question: why are levels of TF elevated in CTEPH? As mentioned above, TF may participate in the pathogenesis of CTEPH by triggering the extrinsic coagulation pathway. The level of TF in monocytes may increase in various pathological conditions such as infection, inflammation, thrombosis, and cancer [17, 29, 30]. In this study, we found that the expression of inflammatory factors (e.g. TNF-*α*, CRP, MCP-1) increased and significantly correlated with TF antigen content in CTEPH. This implies that the expression of TF may be upregulated by these inflammatory factors and it may be of clinical value to detect the levels of these factors in CTEPH patients to provide further information on their condition and prognosis.

Generally, CRP, TNF-α and MCP-1 may be involved in the process of CTEPH pathogenesis by mediating the inflammatory process, which induces the expression of TF in monocytes and aids in the formation of blood clots. An increase in monocyte TF could be disastrous because inflammatory cells, which are regulated by a variety of inflammatory mediators, can not only enhance the expression and activity of TF but can also lead to the injury of vascular endothelial cells. With injury to vascular endothelial cells, increasing amounts of TF are exposed to the blood, which activates the extrinsic coagulation pathway and causes thrombosis formation and deposits in the blood vessel walls. As a consequence, blood vessels become narrow and produce large amounts of inflammatory mediators, resulting in further inflammation. Inflammatory cytokines can induce TF expression and activate the coagulation system, giving rise to the formation of the inflammation-coagulation-thrombosis cycle. There is mounting evidence supporting the existence of such a positive feedback/reversible loop in a complete coagulation-inflammation cycle [31]. For example, TNF-α upregulates the expression of TF in acute respiratory distress syndrome (ARDS) [32], and CRP drastically increases TF expression [33]. Long pentraxin-3, an acute inflammatory molecule, upregulates TF expression in lung injuries [34]. Conversely, guggulsterone (an anti-inflammatory phytosterol) inhibits TF expression and arterial thrombosis [35], adding further proof of inflammation-triggered coagulation. Furthermore, the TF hypercoagulable state results in progressive inflammation as a result of continuous refueling of the coagulation-inflammation cycle. Thrombosis and inflammation are two major pathogeneses of CTEPH, between which there is cross-talk and cooperativity [36]. The inflammation-thrombosis connection provides an alternative pathway through which blood coagulation can indirectly contribute to thrombosis through inflammation, and both processes together promote CTEPH pulmonary vascular remodeling [37]. Currently, few reports have focused on the role of monocyte TF and CTEPH. Our study involved a small number of patients and did not examine the mechanism of TF in CTEPH nor did we compare the change in inflammatory factors before and after treatment. Therefore, further studies with larger patient cohorts that elucidate the molecular mechanism are needed.

## Conclusions

In summary, TF expression was increased in the plasma of patients with CTEPH, partly due to an increase in monocyte TF mRNA levels. Monocyte TF may play an important role during the CTEPH thrombotic process. At the same time, the inflammatory factors CRP, TNF-α and MCP-1 increased in the plasma of patients with CTEPH and correlated with mPAP, indicating that they are involved in the pathogenesis of CTEPH and determine disease severity. Moreover, high expression of TF correlated with expression of the inflammatory factors CRP, TNF-α and MCP-1 in patients with CTEPH. TF, CRP, TNF-α, and MCP-1 may not be attractive molecules to test for screening of CTEPH but may have value in determination of prognosis, which was not evaluated in our study.
